# Supplementary figures and images for: Impact of protein identity on tumor-associated antigen uptake into infiltrating immune cells: A comparison of different fluorescent proteins as model antigens
Source: PLoS One. 2022 Aug 17;17(8):e0272857. doi: 10.1371/journal.pone.0272857 (PMC9384993; doi:10.1371/journal.pone.0272857)

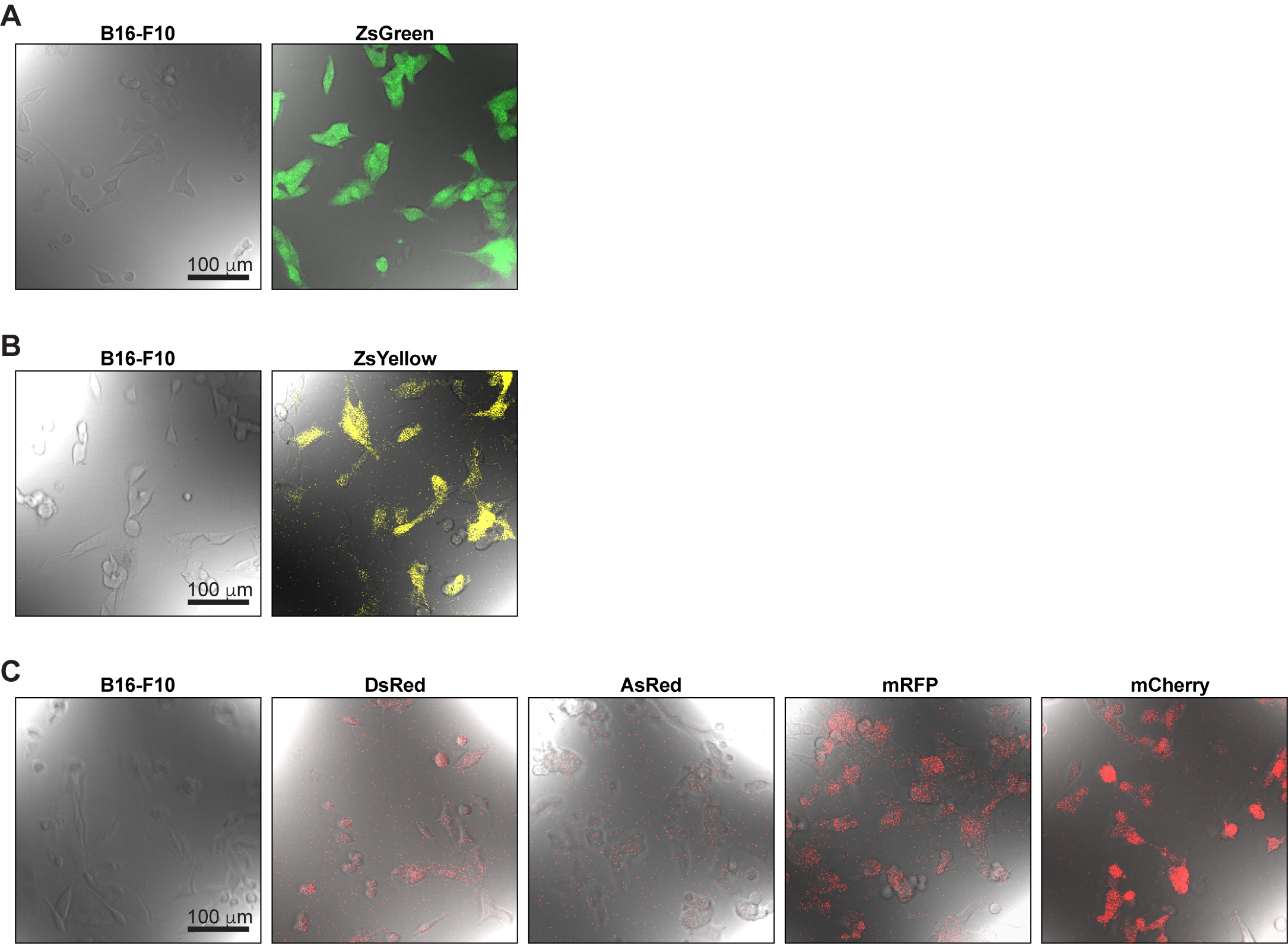

Supplement: S1 Fig — Generated B16 melanoma cells with expression of fluorescent protein ZsGreen (A), ZsYellow (B), AsRed, DsRed, mRFP, and mCherry (all C) respectively, overlayed with their bright field images. Images of B16-F10 cell lines were taken to set background fluorescence. (TIF) [file pone.0272857.s001.tif]

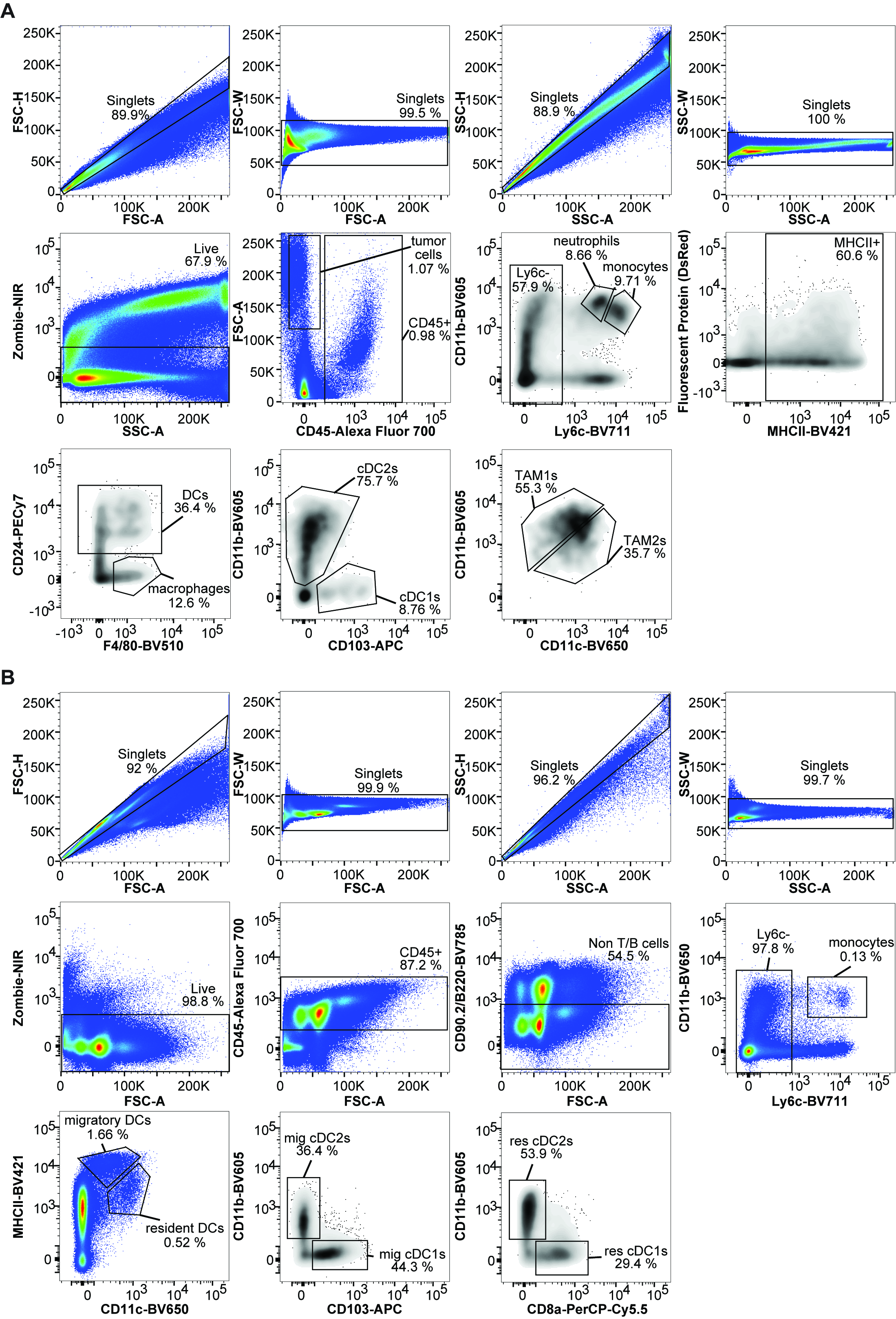

Supplement: S2 Fig — A) Cells collected from tumors were analyzed by flow cytometry using the shown gating methods. Percentages of FP positive cells were analyzed in cDC1, cDC2, TAM1, TAM2, monocytes and neutrophils. B) Lymph node cells were analyzed using these gating methods. The dump- populations are B220, CD 90.2, NK 1.1 lineage marker negative cells. Percentage of FP positive cells were analyzed in migratory cDC1, migratory cDC2, resident cDC1, resident cDC2 and monocytes. (TIF) [file pone.0272857.s002.tif]

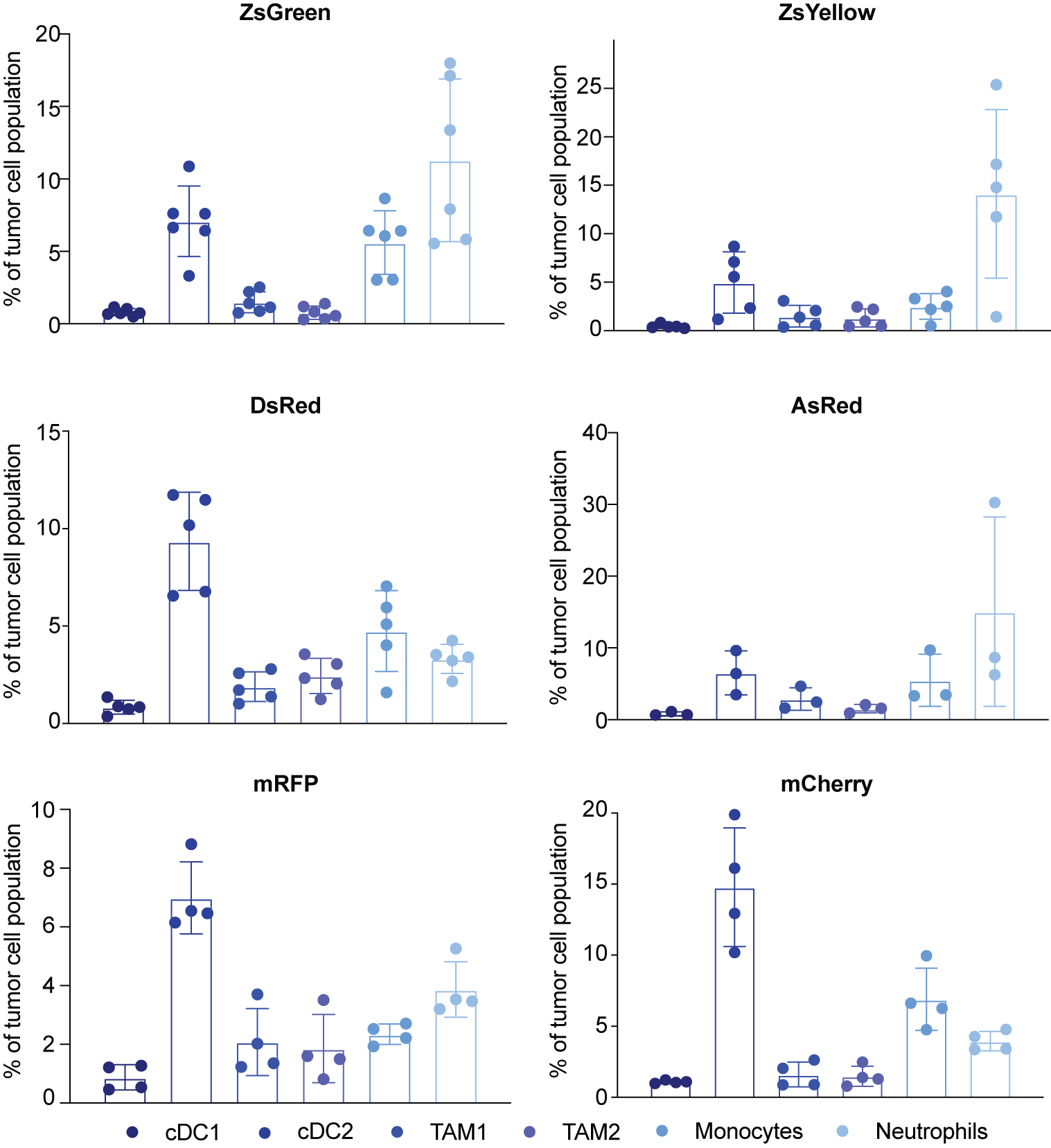

Supplement: S3 Fig — All tumor cells collected were analyzed for their respective immune cell markers. The cell count for each type of immune cell is then plotted as percentages over the total number of live CD45+ cells. (TIF) [file pone.0272857.s003.tif]

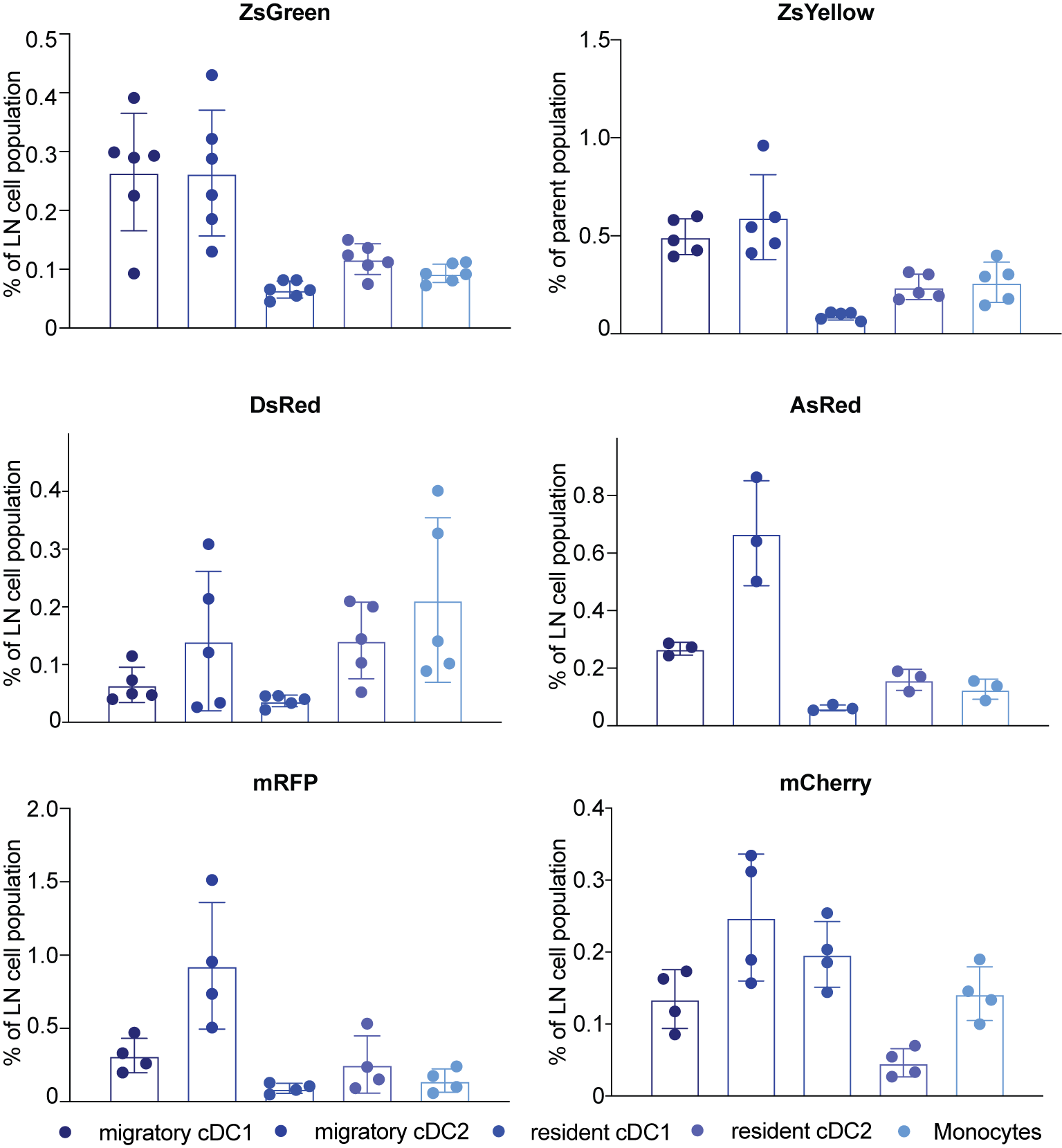

Supplement: S4 Fig — All lymph node cells collected were analyzed for their respective immune cell markers. The cell count for each type of immune cell is then plotted as percentages over the total number of live CD45+ cells. (TIF) [file pone.0272857.s004.tif]
