# Supplementary material for: Impact of protein identity on tumor-associated antigen uptake into infiltrating immune cells: A comparison of different fluorescent proteins as model antigens
Source: PLoS One. 2022 Aug 17;17(8):e0272857. doi: 10.1371/journal.pone.0272857 (PMC9384993; doi:10.1371/journal.pone.0272857)
Supplement: Attachment — Submitted filename: Reponse to Reviewers.pdf [file pone.0272857.s005.pdf]

Response to Reviewers

Thank you all very much for your time and enthusiasm for the data and the conclusions we presented. We find your comments very helpful to provide us a path toward publication in PLOS ONE. Below you will find detailed responses to the reviewer comments.

We want to mention that we also added two additional references which are not responses to reviewers. Since the initial submission in November 2021, a new paper showed that neutrophils can transport bacterial antigens to the lymph nodes in a Ccr7-dependent manner (Özcan et al., Science Immunology (2022), 9126, 7(68)). We think this piece of information is important as the study that we cite for the transport of tumor antigens to the lymph node did not completely rule out neutrophils as a possible transport vehicle. Hence, we modified the text and included the above-mentioned citation and the original citation showing transport of bacterial antigens to the lymph nodes:

> "…Migratory DCs, ~~and~~ monocytes, and neutrophils ~~are~~ can~~responsible for the~~ transport~~ of tumor-associated~~ antigens to the draining ~~td~~LN in a Ccr7-dependent manner [3,14,15].~~,~~ ~~whereas r~~ Resident DCs can receive those antigens through synaptic transfer [5].

Reviewer's comments

Reviewer #1:

1. Is the manuscript technically sound, and do the data support the conclusions?
Reviewer #1: Yes

2. Has the statistical analysis been performed appropriately and rigorously?
Reviewer #1: Yes

3. Have the authors made all data underlying the findings in their manuscript fully available?
Reviewer #1: Yes

4. Is the manuscript presented in an intelligible fashion and written in standard English?
Reviewer #1: Yes

5. Review Comments to the Author
Reviewer #1: Summary: In their report, Serwas et al. conduct a systematic head-on comparison of six fluorescent proteins with a variety of optical features, for their ability to be employed for tumor-assisted antigen uptake studies. While a range of prior reports have independently evaluated these features and unanimously suggested the benefits of ZsGreen, their head-on comparison is unprecedented. This study is important in assisting the choice of the right fluorescent protein depending on the type of study being conducted. The authors report the generation of cell lines with the six fluorescent genes, followed by the evaluation of their in vitro stability. Eventually the genetically modified cell lines are introduced in vivo, and the tumors and the tumor draining lymph nodes are harvested and evaluated for the immune cell population using flow cytometry.

Review:
The paper is scientifically sound and thoughtfully presented. The observation of two different patterns of fluorescent protein-antigen loading in the cells infiltrating the tumors is interesting while the mechanism remains unknown. Notably, all the fluorescent proteins show a similarity in the relative excess of the tumor-associated antigens in cDC2, and tumor associated macrophages. The

experimental work is well done and presented clearly, with the authors highlighting the limitations and proposing the potential alternatives. This novel study opens interesting perspectives for the choice of fluorescent proteins for studying specific antigen uptake pattern. The authors do a good job on reporting the experimental outcomes and logistically address the pros/cons of each evaluated protein. The reviewer particularly appreciates the discussion of the methodology used for gating in flow cytometry section. This is a good practice and is hopefully normalized. The referencing is appropriate and well-formatted. I recommend its publication in PLOS One in its current form.

We thank the reviewer for the positive comments on our manuscript.

Minor comments:

1. The authors need to confirm if the mode of transfection was through retroviral or lentiviral. There seems to be a discrepancy in the text and figure legend.

Thank you for noticing this. The mode of transfection was indeed retroviral, and we changed that accordingly. We also took the chance to assess the correct use of the terms transduction and transfection and correct the language were needed.

2. The authors are encouraged to fix the minor typos in the document.

We have had multiple proof-reading sessions and believe that we have found and corrected all remaining typos.

3. The authors allude to the possibility of certain fluorescent proteins degrading prior to being up taken in the lymph nodes. However, the reviewer wonders if this could also be seen as a function of the changes in fluorescence in the variety of environments they are exposed to.

In theory, the different fluorescent proteins (FPs) should all experience a similar intracellular environment. However, our data shows that there are substantial differences of protein uptake and maintenance dependent on protein identity. Thus, it might indeed be possible that the FPs are shuttled into distinct organelles, and hence, experience a different intracellular environment. We included a short sentence referring to that possibility in our manuscript:

"…Other FPs showed only a limited signal suggesting that proteins could have already been degraded on their way to the draining lymph node, perhaps due to shuttling of the FPs into a different intracellular environment or organelle…"

Reviewer #2:

1. Is the manuscript technically sound, and do the data support the conclusions?
Reviewer #2: Partly

2. Has the statistical analysis been performed appropriately and rigorously?
Reviewer #2: Yes

3. Have the authors made all data underlying the findings in their manuscript fully available?
Reviewer #2: Yes

4. Is the manuscript presented in an intelligible fashion and written in standard English?
Reviewer #2: Yes

5. Review Comments to the Author

Reviewer #2: In this manuscript, authors systematically compared six different fluorescent proteins, namely ZsGreen, ZsYellow, DsRed, AsRed, mCherry, and mRFP. Stability of fluorescent signal was assessed in vitro over time, across different pH environments, and in vivo through FP antigen uptake and transfer to immune cells isolated from tumors and tumor draining LNs. The mechanism of this process is unclear, which made the paper is not comprehensive and convincing. It is reasonable to conduct computational simulation and simply structure alignment to investigate the antigen uptake of those FPs.

We thank the reviewer for critically assessing the quality of our study. We agree with the reviewer that the underlying mechanisms for transfer of FPs, or more generally speaking tumor-associated antigens, to immune cells is not well understood. Previous work suggest that myeloid cells engulf dying tumor cells and ingest them before preparing containing proteins for presentation on their immune cells (Broz et al., Cancer Cell (2014), 638-52, 26(5); Salmon et al., Immunity (2016), 924-38, 44(4); Maier et al., Nature (2020), 257-62, 580(7802)). However, there are a multitude of ways how particles can be ingested into cells (Kumari et al., (2010), 256-75, 20(3)).
The exact consequences of the mode of transfer are not well understood but might be potentially important to determine the elicited immune response. As such we demonstrated in previous work that transferred tumor antigen can be traded among immune cells in the draining lymph nodes (Ruhland et al., Cancer Cell (2020), 786-799, 37(6)). To fully elucidate the underlying mechanism of transfer, and to define consequences of antigen transfer, it is tremendously important to choose the right model antigen. We believe our paper will assist researchers to make the right choice of fluorescent protein. Our findings are important to consider when choosing model antigens for the analysis of antigen uptake and transfer.

The reviewer requests us to undertake a crystallography study:
For us, it is not clear how simple structure alignment and computational simulations should elucidate the mechanism of differential antigen transfer. Only 3 of the chosen fluorescent proteins have available 3D structures on the protein data bank, namely ZsGreen, DsRed and mCherry. The protein structure of fluorescent proteins is quite conserved, so it is not surprising that simple structure alignments with the Matchmaker tool of UCSF ChimeraX (Pettersen et al., Protein Science (2021), 70-82, 30 (1)) result in a substantial overlap of the structures (see Reviewer Figure 1). However, the entire understanding of what will make these structures more or less labile is complex and, we feel, well beyond the goal of this publication which is intended to highlight important differences.

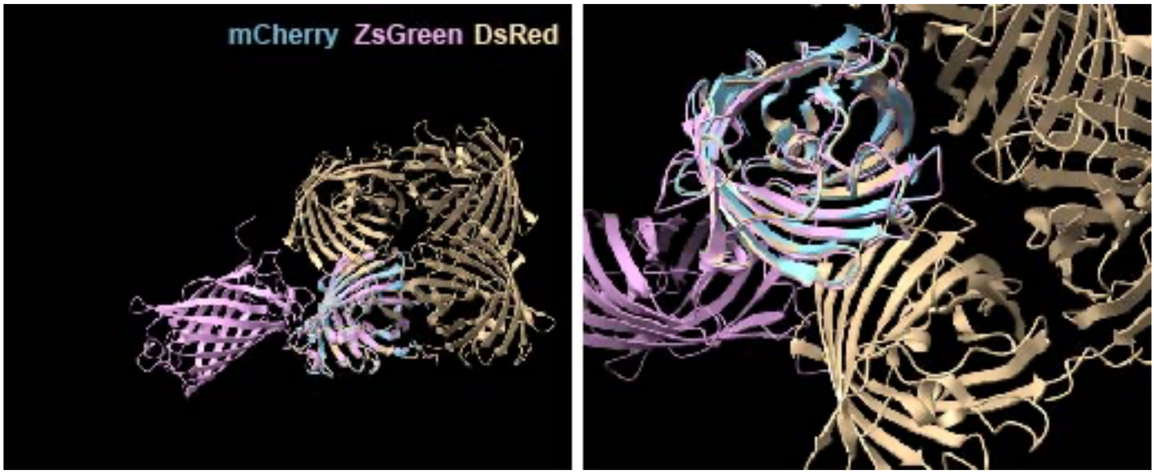

Reviewer Figure 1: Structure Alignment of 3D structures of mCherry (2h5q), ZsGreen (2ojk), and DsRed (1zgo) performed with the Matchmaker tool of UCSF ChimeraX.

At the highest level of abstraction, the main difference between these chosen proteins is the tendency to form dimers, tetramers, or remain as monomers. It is not clear whether these changes result in different antigen transfer, uptake, and/or maintenance, but could be a possibility. We added a sentence, and a corresponding reference, reflecting the different tendencies for multimerization of the proteins into our manuscript:

"…It remains unclear what causes the increased percentage of FP-positive tumor infiltrating cDC1s in mRFP and mCherry-expressing tumors. Numbers of cDC1s are not different between tumors expressing FPs irrespectively of whether they cause 'classical' or 'non-canonical' antigen uptake patterns (S3 Fig). Of note, whereas ZsGreen, ZsYellow, AsRed, and DsRed are proteins directly isolated from distinct organism, mCherry and mRFP have been designed to better serve as tools for molecular biology. Changes aimed to enhance brightness and stability, and to prevent dimerization [10,13]. ..."
